# Minimized Bolus-Type Wireless Sensor Node with a Built-In Three-Axis Acceleration Meter for Monitoring a Cow’s Rumen Conditions

**DOI:** 10.3390/s17040687

**Published:** 2017-03-27

**Authors:** Hirofumi Nogami, Shozo Arai, Hironao Okada, Lan Zhan, Toshihiro Itoh

**Affiliations:** 1 Faculty of Engineering, Kyushu University, Fukuoka 819-0395, Japan; 2 Research Center for Ubiquitous MEMS and Micro Engineering (UMEMSME), Advanced Industrial Science and Technology (AIST), Tukuba 305-8564, Japan; hironao.okada@aist.go.jp (H.O.); chou-ran@aist.go.jp (L.Z.); toshihiro-itoh@aist.go.jp (T.I.); 3 National Institute of Animal Health, National Agriculture and Food Research Organization (NARO), Tukuba 305-0856, Japan; sarai@affrc.go.jp; 4 Graduate School of Frontier Sciences, The University of Tokyo, Kashiwa 277-8563, Japan

**Keywords:** animal health monitoring system, rumen monitoring, bolus type wireless sensor node

## Abstract

Monitoring rumen conditions in cows is important because a dysfunctional rumen system may cause death. Sub-acute ruminal acidosis (SARA) is a typical disease in cows, and is characterized by repeated periods of low ruminal pH. SARA is regarded as a trigger for rumen atony, rumenitis, and abomasal displacement, which may cause death. In previous studies, rumen conditions were evaluated by wireless sensor nodes with pH measurement capability. The primary advantage of the pH sensor is its ability to continuously measure ruminal pH. However, these sensor nodes have short lifetimes since they are limited by the finite volume of the internal liquid of the reference electrode. Mimicking rumen atony, we attempt to evaluate the rumen condition using wireless sensor nodes with three-axis accelerometers. The theoretical life span of such sensor nodes depends mainly on the transmission frequency of acceleration data and the size of the battery, and the proposed sensor nodes are 30.0 mm in diameter and 70.0 mm in length and have a life span of over 600 days. Using the sensor nodes, we compare the rumen motility of the force transducer measurement with the three-axis accelerometer data. As a result, we can detect discriminative movement of rumen atony.

## 1. Introduction

For the purpose of increased safety and security, wireless sensor network systems are being used increasingly in applications such as structural health monitoring, human health monitoring, agricultural field monitoring, and animal health monitoring [1,2,3,4,5,6]. Structural health monitoring can improve the safety and reliability of buildings, bridges, tunnels, and express highways by detecting damage before it reaches a critical state. This damage is sensed by wireless sensor nodes installed on the structure [1,2]. Human health monitoring detects sleep disorders, Parkinson’s disease, etc. by the logging of a person’s daily walking movements and posture using Global Positioning System (GPS) devices, triaxial accelerometers, and angular velocity sensors [3]. These technologies have also been introduced in agricultural field monitoring, including animal health monitoring [4,5,6,7]. It is believed that wireless sensor nodes attached to animals, in conjunction with wireless health-monitoring systems, can achieve early detection and prevention of diseases, and thus reduce economic losses. In this study, we focus on developing an animal health monitoring system for cows.

**Figure 1:**
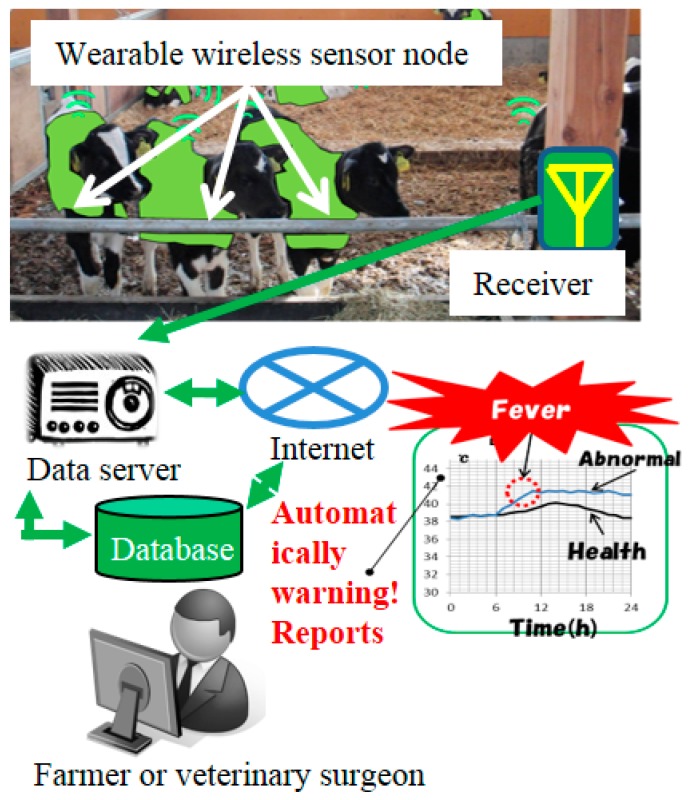
Animal health monitoring system for cows.

Figure 1 shows the concept of our health monitoring system to automatically detect sick cows in the farm. In this system, sensor nodes attached to cows measure their body temperature and activity. If one wireless sensor node transmits an abnormal temperature or activity, the system logs the data on the collected sickness pattern, and automatically sends out a warning signal. Furthermore, the system compares the logged data with the expected normal data from a cow, and determines the causes of the abnormal reading [8,9]. Other studies have developed a wearable wireless estrus detection sensor [10] or a portable estrus intensity detection sensor [11]. In this study, to monitor rumen conditions in adult cattle, we fabricated bolus type wireless sensor nodes.

It is important to monitor rumen conditions in cows, as a dysfunctional rumen system may cause death. Sub-acute ruminal acidosis (SARA) is common in cows, and is characterized by repeated periods of low ruminal pH [12]. SARA has been reported in 26% of early-lactation and mid-lactation cows and in 10%–15% of grazing cows [13]. SARA causes other diseases, including ruminitis, abomasal displacement, ruminal tympany, and so on [14]. Thus, to monitor ruminal pH, Sato developed bolus-type wireless sensor nodes [15,16]. The sensor nodes were inserted into the rumen using a special instrument via the cow’s oral cavity. The ruminal pH was measured and this information was then transmitted wirelessly. The sensor can be used continuously for a period of two to three months without replacing the battery, and can be recovered orally with special instruments. The primary advantage of the sensor is its ability to continuously measure ruminal pH and estimate rumen conditions. However, the volume of the internal liquid in the comparison electrode limits the life of the sensor, as it is used up during pH measurements. Therefore, we use a new three-axis accelerometer sensor to estimate rumen conditions, whose lifespan is significantly longer.

**Figure 2:**
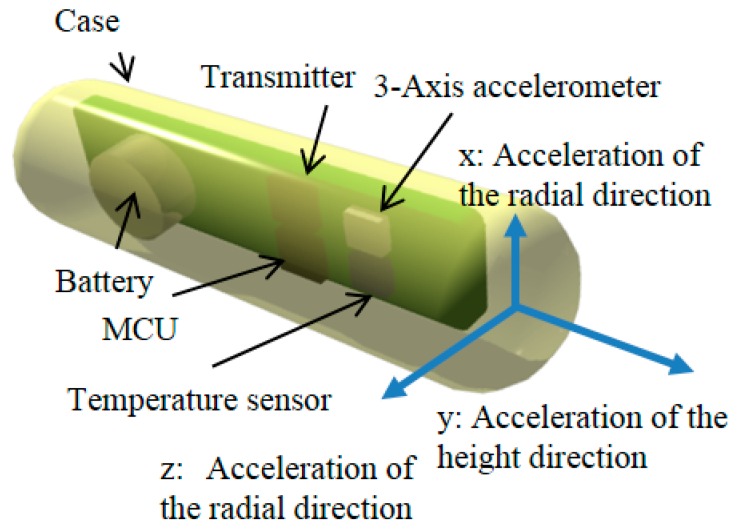
Model of a bolus-type sensor node

Figure 2 shows the model of our bolus-type sensor nodes. These sensor nodes has a three-axis accelerometer and a temperature sensor, and sensor substrates are fixed on the inside case. Thus, the X-unit and Z-unit indicates the radial acceleration of the cylinder, and Y-units indicate vertical acceleration. When the rumen contracts, it is assumed that the sensor nodes move with the ruminal contents. By plotting the movement of the ruminal contents, we can gauge the health of the rumen system.

In this paper, we fabricated proto-wireless sensor nodes. We compared the rumen motility of the force transducer measurement [17,18] with the three-axis accelerometer. In this experiment, we administered xylazine hydrochloride (a muscle relaxant) intra-muscularly to simulate the rumen-stopping condition, and this served as a quasi-test of rumen atony. We also compared the wireless sensor nodes data (three-axis acceleration-meter data and temperature data) with the behavior of the cow. The behavior was eating (ruminating), non-eating, drinking, standing, and lying.

## 2. Materials and Methods

### 2.1. Design of the Wireless Sensor Node

**Figure 3:**
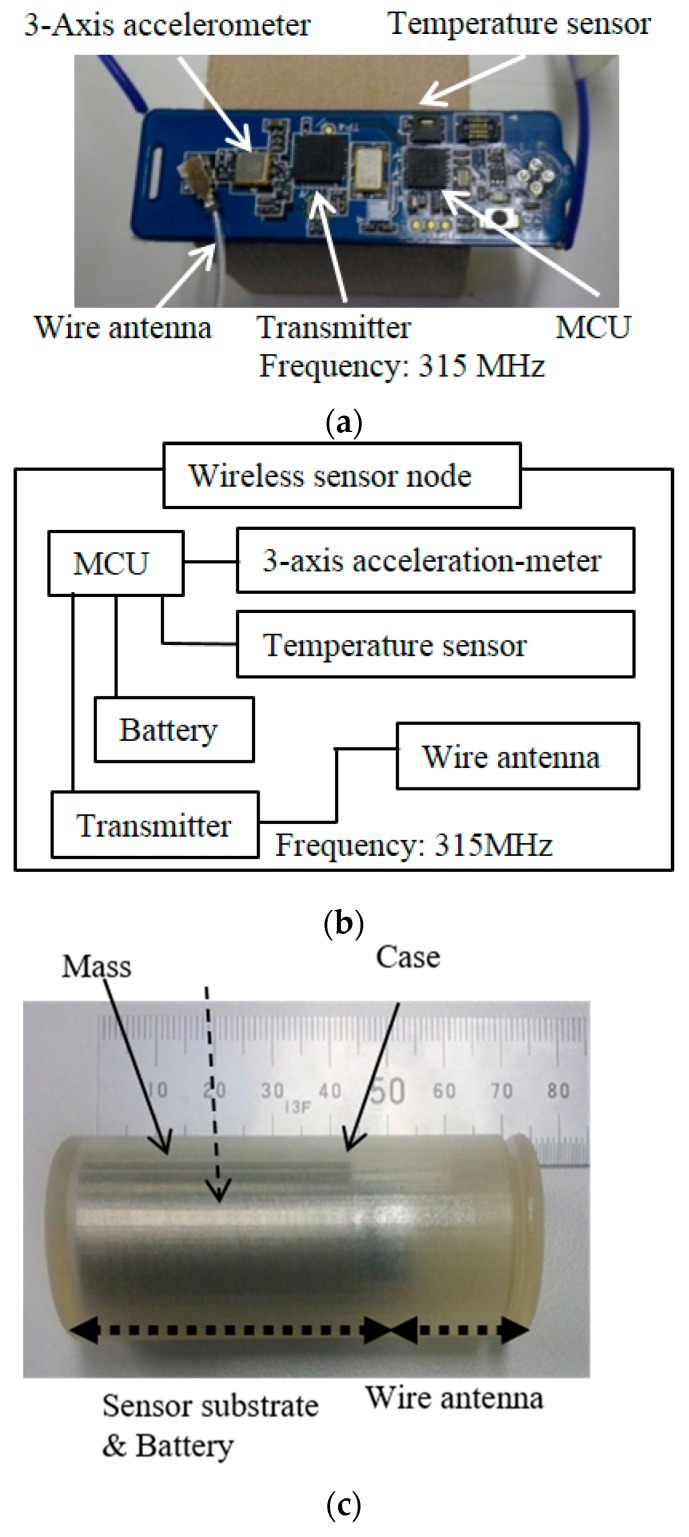
Photograph of the sensor substrate (**a**); a block diagram of it (**b**); and a photograph of our bolus-type wireless sensor node (**c**).

We designed wireless sensor nodes that can be administered orally. For this purpose, the sensor nodes are cylinder-shaped and consist of the sensor node substrate, the mass, the battery, and the antenna. Figure 3a,b shows a photograph and the block diagram of the sensor node substrates. The size of the substrate is 15.0 mm × 47.4 mm × 2.3 mm and it weighs 2.0 g. The 3-axis accelerometer, temperature sensor, micro control unit (MCU), and transmitter were mounted on the substrate. The operating frequency of this transmitter was 315 MHz because the permeability of radio waves at this frequency is high for animals. The communication distance was over 20 m without obstacles. The sensors used a wire antenna whose distance was about 24 cm. The measurement and transmission interval was set from 0.5 s to 120 s.

Figure 3c indicates the wireless sensor nodes. The case (the cylinder and the cap) was made of ultraviolet curable resin using a 3D printer. The sensor nodes consist of the sensor substrate, battery, and wire antenna. The sensor substrate and battery part includes the mass. The mass controls the weight of the sensor nodes to be placed into the rumen. If the sensor nodes are too light, the sensor nodes will be brought up when chewing the cud (ruminating), leading to loss and/or damage. The wire antenna is set on the edge-cylinder. To obtain a high gain, the wire antenna is shaped in the form of a spiral. These parts are put inside the case, which is sealed with an adhesive.

The sensor nodes are 30.0 mm in diameter and 70.0 mm in length, and have a density of 1.9 g/cm^3^. Their cylindrical shape and size allow oral administration because they are smaller (30 mm in diameter and 145 mm in length) than sensors reported in a previous study [15,16].

### 2.2. Experimental Setup

**Figure 4:**
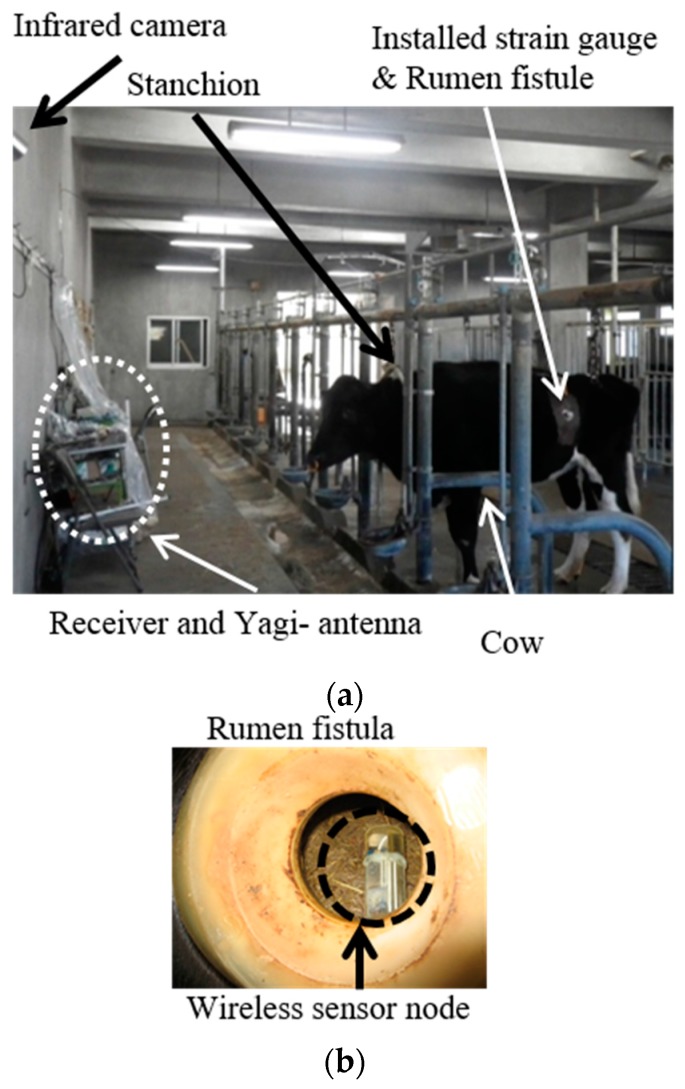
Experimental environment (**a**) and the rumen fistula (**b**).

We performed the experiments on one cow. Figure 4a shows the experimental environment. The strain gauges and rumen fistula were installed surgically. The strain gauges were attached to the outside of the rumen and can detect rumen motility. This method is called force transducer measurement [17,18]. The rumen fistula is the artificial window attached to the rumen, which is useful method for digestion study. Our sensor node was put in the rumen from the rumen fistula (Figure 4b). The 3-axis acceleration-meter and temperature data of the sensor node were collected with the receiver and the yagi-antenna. The measurement distance was kept nearly constant at about 1.5 m, because the cow could be standing or sitting, but not moving as it is locked with the stanchion. We could confirm cow behavior, as eating (ruminating), non-eating, drinking, standing, and lying by using an infrared camera, which was connected to the notebook computer and recorded the movie.

In the beginning, we examined the possibility of detecting rumen atony, which is a rumen-stopping condition. The rumen-stopping condition was caused by administering xylazine hydrochoride intra-muscularly. We compared the rumen motility of the force transducer measurement with the 3-axis acceleration-meter data of our wireless sensor node. Next, we compared the wireless sensor nodes data (3-axis acceleration-meter data and temperature data) with the behavior of the cow. The behavior was eating (ruminating), non-eating, drinking, standing, and lying.

## 3. Results

### 3.1. The Quasi-Test of the Rumen Atony

**Figure 5:**
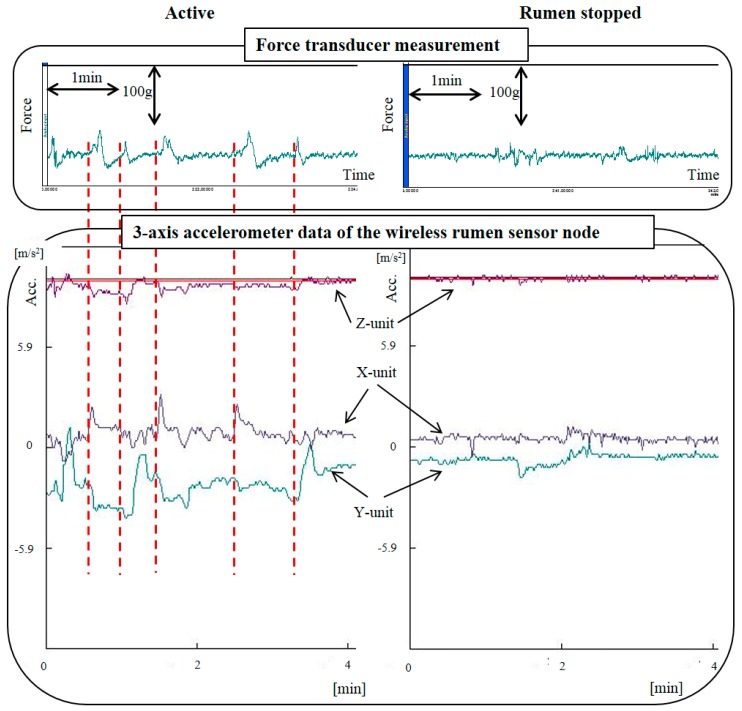
The top part shows the result of the force transducer measurement, while the bottom part shows the result of the three-axis accelerometer data of the wireless sensor node.

Figure 5 shows the measurement results. The top part shows the result of the force transducer measurement, while the bottom part shows the result of the three-axis accelerometer data of the wireless sensor node. The left side corresponds to a rumen active condition and the right side corresponds to when the rumen had stopped. The rumen-stopped condition was the quasi-test for rumen atony, and was produced by administering xylazine hydrochloride intramuscularly. When the rumen was in an active condition, the contractile force could be observed about twice per 1 min (upper left graph, Figure 5). We allowed the simultaneous monitoring of the change of acceleration of the rumen sensor node (lower left graph). Any one of the x-unit, y-unit, and z-unit readings indicates a big change in the acceleration. In the rumen-stopped condition, the contractile force could not be generated (upper right graph). Similarly, the change in acceleration could not be generated (lower right graph). From these results, it is seen that our sensor nodes can detect signs of rumen atony.

### 3.2. Compared Cow Behavior with Wireless Sensor Nodes Data

**Figure 6:**
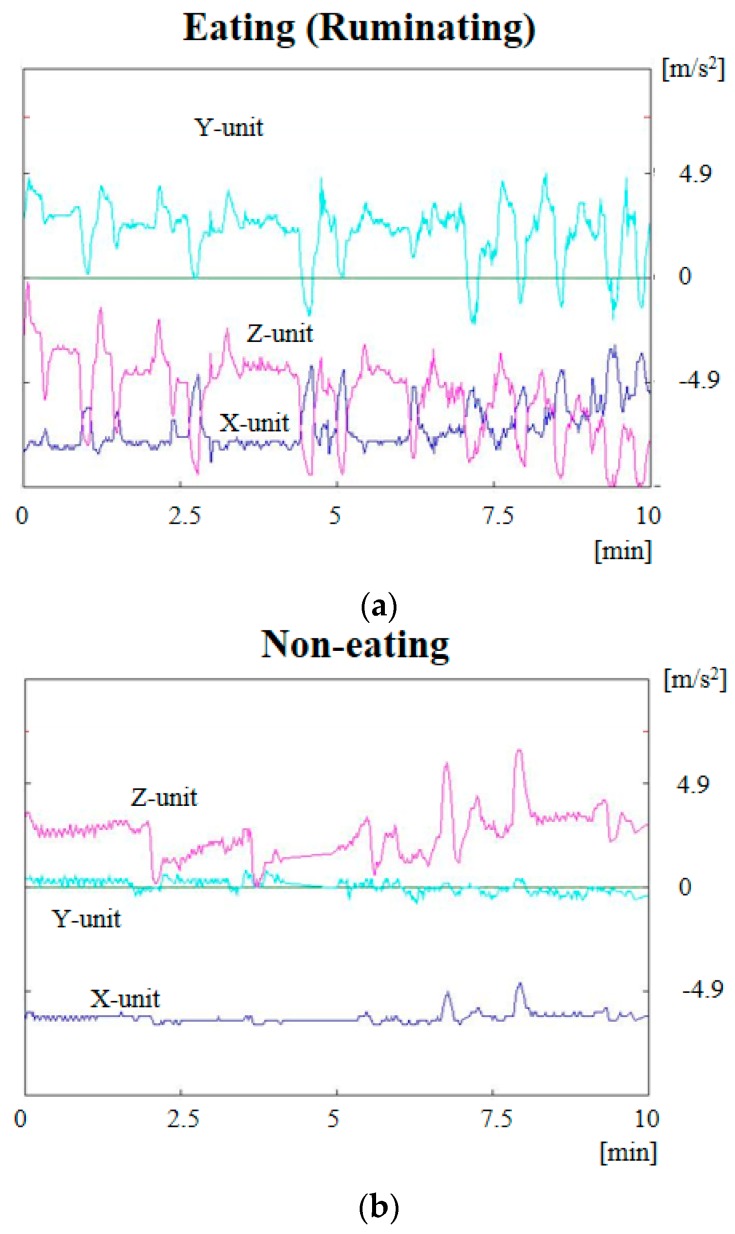
Measurement results of the change of acceleration data for x-axis, y-axis and z-axis within 10 min when the cow is eating (ruminating) (**a**) and when the cow is non-eating (**b**).

Figure 6 indicates the measurement results of the change in acceleration for x-axis, y-axis and z-axis within 10 min. The acceleration of the y-unit is the vertical direction of the cylinder, and the acceleration of the x-unit and z-unit is the radial direction of the cylinder. Figure 6a shows the situation when the cow is eating (ruminating), and Figure 6b shows the situation when it is non-eating. All the three-axis accelerometers indicate big changes in 1 min or 30 s intervals. On the other hand, the three-axis acceleration changed slightly when the cow was non-eating. From these results, it is possible that the sensor nodes can distinguish activity level.

**Figure 7:**
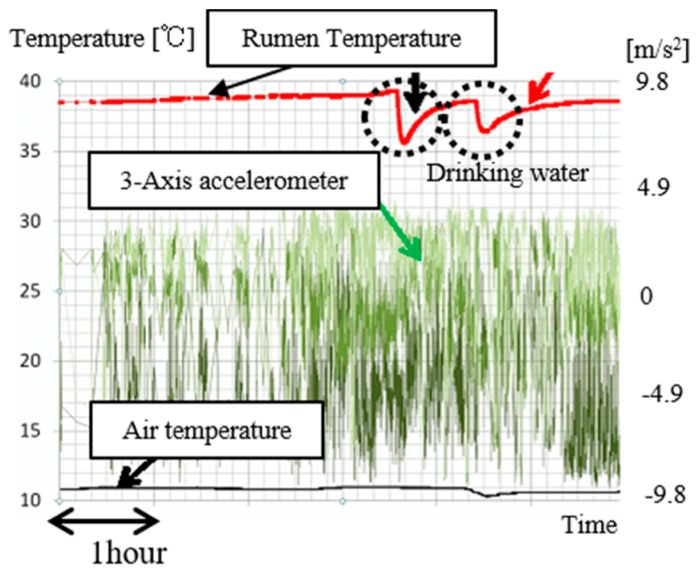
Temperature data and three-axis acceleration data for x-axis, y-axis and z-axis when the cow was drinking.

Figure 7 shows temperature data and three-axis acceleration data when the cow was drinking. The temperature of the sensor node usually indicates a stable state when the temperature is between 38 °C and 40 °C. When the cow was drinking water, the temperature temporarily decreased at a fast rate and then rose slowly. It is seen that drinking behavior can also be detected.

**Figure 8:**
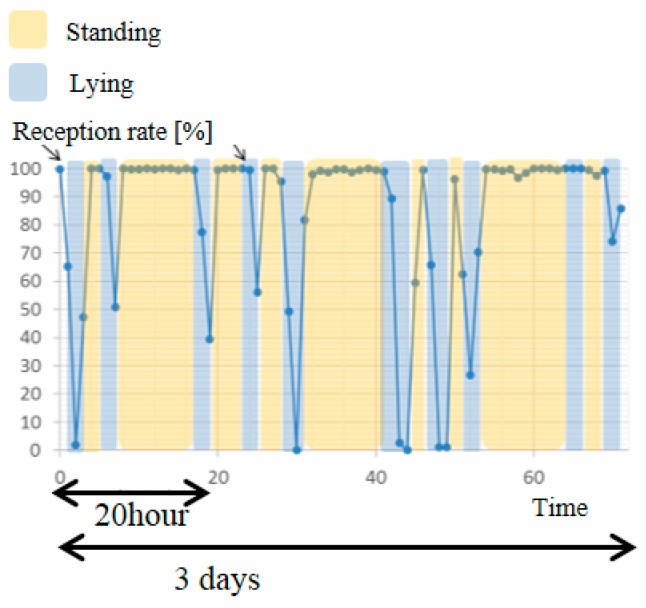
Reception rate per hour for 3 days of wireless sensor nodes.

Figure 8 indicates the reception rate per hour for 3 days. The reception rate was almost over 90%, but occasionally fell below 90%. Using the movie recorded by the infrared camera, we confirmed the position of the cow. The orange area was the standing position and the blue area was the lying position. It can be seen that the reception rate decreased when the cow was sitting. In the future, it will be necessary to take effective measures to control the decreasing reception rate.

## 4. Discussion

Our sensor nodes can be used for the early detection of rumen atony as shown in Figure 5. The quasi-test of rumen atony indicated that rumen contents were stopped when the cow stopped ruminating.

Additionally, from Figure 5 and Figure 6, it is seen that it is possible to determine rumen conditions. When the rumen contracts, a large force variation from the force transducer can be used to evaluate the number and the contractile force. Similarly, the acceleration of the wireless sensor node can also be synchronized. The acceleration may be inferred by the contractile force and the ruminating frequency. In particular, the rumen in the ruminating state was actively moving, while it was not moving when the cow was not-eating. From these results, it is possible that the sensor nodes can distinguish activity level.

As shown in Figure 8, the lying position decreased the reception rate. The distance between the sensor node and the antenna did not change between sitting and standing, as the cow was fixed to the stanchion. Therefore, the cause of the decreasing reception rate may be the attenuation of radio waves because of the proximity to the ground. In the future, it is necessary to take effective measures to control the decreasing reception rate. For example, the transmission power increases when the cow is lying. We assume that the data is pooled to the logger when the cow is lying and transmitted when the cow is standing. In addition, we need to investigate the cause of decreasing reception rate when the cow is lying.

## 5. Conclusions

We successfully fabricated a minimized wireless sensor node capable of monitoring long-term rumen conditions. The sensor nodes enable us to measure three-axis acceleration and temperature. The sensor nodes were cylinder-shaped with a diameter of 30.0 mm and a length of 70 mm. With an optimum measurement and transmission frequency of 0.2 Hz and a power consumption of 0.18 mA, the theoretical life-span of the sensor is over 600 days. Using the sensor nodes, we compared the rumen motility of the force transducer measurement with the three-axis accelerometer data. As a result, we can detect discriminative movement of rumen atony.
